# The modulation of somatosensory resonance by psychopathic traits and empathy

**DOI:** 10.3389/fnhum.2013.00274

**Published:** 2013-06-19

**Authors:** Louis-Alexandre Marcoux, Pierre-Emmanuel Michon, Julien I. A. Voisin, Sophie Lemelin, Etienne Vachon-Presseau, Philip L. Jackson

**Affiliations:** ^1^École de Psychologie, Université LavalQuébec, QC, Canada; ^2^Centre de Recherche de l'Institut Universitaire en Santé MentaleQuébec, QC, Canada; ^3^Centre Interdisciplinaire de Recherche en Réadaptation et Intégration SocialeQuébec, QC, Canada; ^4^Département de Réadaptation, Université LavalQuébec, QC, Canada

**Keywords:** pain perception, psychopathic traits, somatosensory resonance, shared representations, empathy

## Abstract

A large number of neuroimaging studies have shown neural overlaps between first-hand experiences of pain and the perception of pain in others. This shared neural representation of vicarious pain is thought to involve both affective and sensorimotor systems. A number of individual factors are thought to modulate the cerebral response to other's pain. The goal of this study was to investigate the impact of psychopathic traits on the relation between sensorimotor resonance to other's pain and self-reported empathy. Our group has previously shown that a steady-state response to non-painful stimulation is modulated by the observation of other people's bodily pain. This change in somatosensory response was interpreted as a form of somatosensory gating (SG). Here, using the same technique, SG was compared between two groups of 15 young adult males: one scoring very high on a self-reported measure of psychopathic traits [60.8 ± 4.98; Levenson's Self-Report Psychopathy Scale (LSRP)] and one scoring very low (42.7 ± 2.94). The results showed a significantly greater reduction of SG to pain observation for the high psychopathic traits group compared to the low psychopathic traits group. SG to pain observation was positively correlated with affective and interpersonal facet of psychopathy in the whole sample. The high psychopathic traits group also reported lower empathic concern (EC) scores than the low psychopathic traits group. Importantly, primary psychopathy, as assessed by the LSRP, mediated the relation between EC and SG to pain observation. Together, these results suggest that increase somatosensory resonance to other's pain is not exclusively explained by trait empathy and may be linked to other personality dimensions, such as psychopathic traits.

## Introduction

Does vicariously experiencing someone else's pain help us understand and care about the distress this person might be feeling? Over the last decade, a large number of studies in healthy and clinical populations have used the representation of other people's pain as a means to investigate the different dimensions of empathy. The construct of empathy can be defined as the capacity to be in tuned with the affective experience of someone else. It involves, beyond a cognitive effort to understand and imagine someone else's state, a disposition to emotionally identify with other's feeling and to share their affective experience (Decety and Jackson, 2004; Kernberg, 2012a). Accordingly, this suggests that, at the brain level, multimodal neural networks are at play during empathic response. Early neuroimaging studies on pain observation have revealed a considerable overlap between cerebral regions involved in the direct experience of pain and its perception in others (e.g., Morrison et al., 2004; Singer et al., 2004; Jackson et al., 2005), suggesting the existence of a neuronal pathway implicated in the elaboration of representations that reflect our own responses to pain to understand how the pain of others feels (see Jackson et al., 2006; Lamm et al., 2011 for reviews). This shared neural representations between the perception of pain in self and other has been interpreted as the result of an automatic resonance mechanism (Jackson et al., 2006) that can be best described as the lower-level of a vicarious pain response on which higher order process operate to develop empathy (Han et al., 2009; Vachon-Presseau et al., 2011).

From initial clinical descriptions to contemporary taxonomies, psychopathy has been prototypically associated with severe emotional disturbances and empathy breakdown (Cleckley, 1941; Lilienfeld and Andrews, 1996; Hare, 2003). This personality disorder is better understood as a constellation of personality traits that encompass affective and interpersonal qualities along with behaviors reflecting a socially deviant lifestyle (Hare, 2003). Primary psychopathy has been designated as the heritable traits of emotional detachment commonly reported as a lack of compassion and guilt, callous misuse of others for personal gain and failure to form close interpersonal attachment (Levenson et al., 1995; Poythress and Skeem, 2006). Secondary psychopathy usually refers to poor behavioral control, hostility and antisociality (Levenson et al., 1995). The majority of research on psychopathy has focused on samples of incarcerated male offenders, which has led to some pending interrogations about the generalizability of these results to community samples (Hall et al., 2004). Still, studies in non-incarcerated samples have gained in popularity, as the dimensional approach to personality disorders has obtained support from both clinical and research fields (for a review on the clinical perspective see Kernberg, 2012b).

Several transcranial magnetic stimulation (TMS; e.g., Avenanti et al., 2005), somatosensory-evoked potentials (SEP; e.g., Bufalari et al., 2007; Martínez-Jauand et al., 2012), magnetoencephalography (e.g., Cheng et al., 2008), functional magnetic resonance imaging (fMRI; e.g., Lamm et al., 2007; Saarela et al., 2007; Han et al., 2009) and somatosensory steady-state response (SSSR; e.g., Voisin et al., 2011a) studies have shown that brain regions processing the sensory dimension of first hand pain (i.e., somatosensory cortices) are also modulated by the observation of visual stimuli depicting body limbs in pain (Voisin et al., 2011a), painful facial expressions (Saarela et al., 2007), and even psychological painful scenarios (e.g., social rejection in Kross et al., 2012). Some studies have also demonstrated that this resonance mechanism can be modulated by individual factors such as state-reactivity (Avenanti et al., 2009), trait empathy (Avenanti et al., 2009; Vachon-Presseau et al., 2011) and callous-unemotional traits (Fecteau et al., 2008). The study of Fecteau et al. (2008), in which a community sample of men was exposed to visual stimuli depicting hands in painful and non-painful scenarios, was the first to show a positive correlation between suppression of motor evoked potentials (MEPs) and the score of their participants on the Coldheartedness subscale of the psychopathic personality inventory (PPI; Lilienfeld and Andrews, 1996). This result seemed counter-intuitive because increase sensorimotor resonance to the pain of others had been positively associated with self-reported empathy (Avenanti et al., 2009). However, it was also suggested that this automatic neural response could trigger distress (Decety, 2011) and threat related networks (Ibáñez et al., 2011), therefore advocating for an alternative or concomitant view to automatic pain resonance that simply implies arousal. This would also support the view that regulation processes of sensorimotor responses are required in order to respond empathically to the pain of others (Han et al., 2009). Together, these results suggest that sensorimotor resonance to the pain of others is not a direct path to empathy and further investigation on the role of psychopathic traits could be useful to better understand this relationship.

One question arising is how psychopathic traits influence the somatosensory resonance mechanisms involved in the perception of pain in others. To date, only one study has investigated the sensorimotor resonance to other's pain in a community sample of men with psychopathic traits (Fecteau et al., 2008). Although this TMS study has revealed intriguing and initially counterintuitive findings, it has mainly focused on the motor aspect of resonance. Previous studies have shown that seeing pain in others reduces somatosensory steady-state response (SSSR) to a non-painful stimulation (Voisin et al., 2011a) and that this reduction is specific to the frequency of the mechanical stimulation, reinforcing the idea that the modulation in SSSR reflects the inhibition (gating) of somatosensory activity by attention (Mayer et al., 2009). In order to gain understanding on the relationship between psychopathic traits and sensory resonance, we measured SSSR of participants exposed to clips depicting pain-evoking or neutral situations.

The aim of the present study was to investigate the somatosensory aspect of the resonance to other's pain in two groups of men selected from a large community: one group scoring very high and one group very low on a psychopathic traits measure [Levenson's Self-Report Psychopathy Scale (LSRP), Levenson et al., 1995]. Another objective of this study was to examine the relationship between the somatosensory response, self-reported empathy, and psychopathy. We used the modulation of the somatosensory response to a mechanical stimulation as a function of the visual stimuli depicting different levels of bodily pain (Voisin et al., 2011a) as a measure of somatosensory gating (SG). This response was subsequently compared with: (1) vicarious pain ratings, (2) the scores on a measure of trait-empathy [Interpersonal Reactivity Index (IRI), Davis, 1980] and (3) the scores on the LSRP (Levenson et al., 1995). We first expected to find lower scores on the affective subscale of the IRI in high psychopathic traits males compared to the low psychopathic traits ones. Taking into account that both the hypotheses of sensorimotor resonance mechanisms (Bufalari et al., 2007; Lamm et al., 2007) and arousal (Decety, 2011) might be at play during pain empathy, we also expected that participants with high psychopathic traits would have a greater SG to pain observation compared to individuals with low psychopathic traits. Finally, according to Fecteau et al. (2008) we posited that SG would be positively correlated with the affective and the interpersonal facets of psychopathy.

## Materials and methods

### Participants

Only males were invited to participate to this experiment because the prevalence of psychopathy in women is much lower than in men (e.g., Salekin et al., 1997; Jackson et al., 2002). One hundred and sixty four undergraduate right-handed male students were recruited across different Faculties of Université Laval, Québec, and asked to complete the LSRP (Levenson et al., 1995; see description below) in class. From this initial sample, and based on the distribution of the LSRP_Total scores, two sub-groups were invited to participate to an EEG protocol: 15 participants in the upper third (LSRP_High), and 15 participants in the lower third (LSRP_Low) (see Table 1 for detailed characteristics of the sample). The LSRP_High total scores (60.8 ± 4.98) were significantly higher than the LSRP_Low scores [42.7 ± 2.94; *t*_(29)_ = 12.12, *p* < 0.001]. Participants reported having no history of neurological, pain-related, or psychiatric disorders, were not taking any medication, and had normal or corrected-to-normal vision. The participants received monetary compensation for their travel expenses to the laboratory and they each gave written informed consent. The study was approved by the Ethics Committees of the research center (CIRRIS-IRDPQ) and Université Laval.

**Table 1:** **Mean age and scores on self-reports of psychopathy**.

**Groups**	***N***	**Age**	**LSRP_Total**	**PP1**	**PP2**
		***X* (*SD*)**	***X* (*SD*)**	***X* (*SD*)**	***X* (*SD*)**
Whole sample	164	22.2 (2.75)	50.9 (6.31)	34.2 (5.6)	18.1 (3.2)
LSRP_Low	15	23.7 (2.9)	42.7 (2.94)^***^	27.7 (4.7)^***^	17.1 (2.9)^**^
LSRP_High	15	22.3 (1.44)	60.8 (4.98)^***^	38.8 (4.3)^***^	20.0 (4.3)^**^

** *p < 0.01*,

*** *p < 0.001*.

*PP1, primary psychopathy subscale; PP2, secondary psychopathy subscale*.

### Measures and materials

#### Questionnaires

The LSRP (Levenson et al., 1995) is a 26-item self-reported measure of psychopathic traits developed for use in community samples. The LSRP assess primary and secondary psychopathy, two factors of the most predominant psychopathic measure, the Hare Psychopathy Checklist (PCL-R; Hare, 2003). Each item consists in a statement that the participant endorses on a 4-point Likert-type scale (1 = *disagree strongly* to 4 = *agree strongly*). The primary psychopathy subscale (PP1) consists in 16 items measuring an inclination to lie, a lack of remorse, callousness, and manipulativeness. The secondary psychopathy subscale (PP2) consists in 10 items measuring impulsivity, frustration tolerance, quick-temperedness, and lack of long-term goals.

The Davis' IRI (Davis, 1980) is a 28-item self-report instrument that assesses trait empathy, that is, one's own reactions to the observation of another's experiences. Each item is rated on a scale ranging from 1 (*does not describe me well*) to 5 (*describes me very well*). The IRI is composed of four subscales thought to reflect the affective and cognitive aspects of empathy: Empathic Concern (EC) and Personal Distress (PD), Fantasy (FS) and Perspective Taking (PT). The EC subscale measures experienced feelings of sympathy and compassion for others in distress. The PD measures self-oriented feelings of anxiety and distress in response to tense interpersonal situations. The FS scale measures the tendency to project oneself into fictional situations. The PT subscale measures the tendency to adopt the psychological point of view of others.

The situational pain questionnaire (SPQ; Clark and Yang, 1983) was used in order to evaluate how participants estimated their own sensitivity to pain. The discrimination scores P(A), indicate the extent to which subjects are able to differentiate painful scenarios from neutral, while the response bias scores B, indicate the degree to which the situations are considered painful (for details on the method see Danziger et al., 2006). The questionnaire consists in 15 events that are considered to be relatively painful and 15 non-painful events. Items are rated by using a numerical scale ranging from 1 (*not noticeable*) to 10 (*worst possible pain*).

#### Visual stimuli

**Figure 1:**
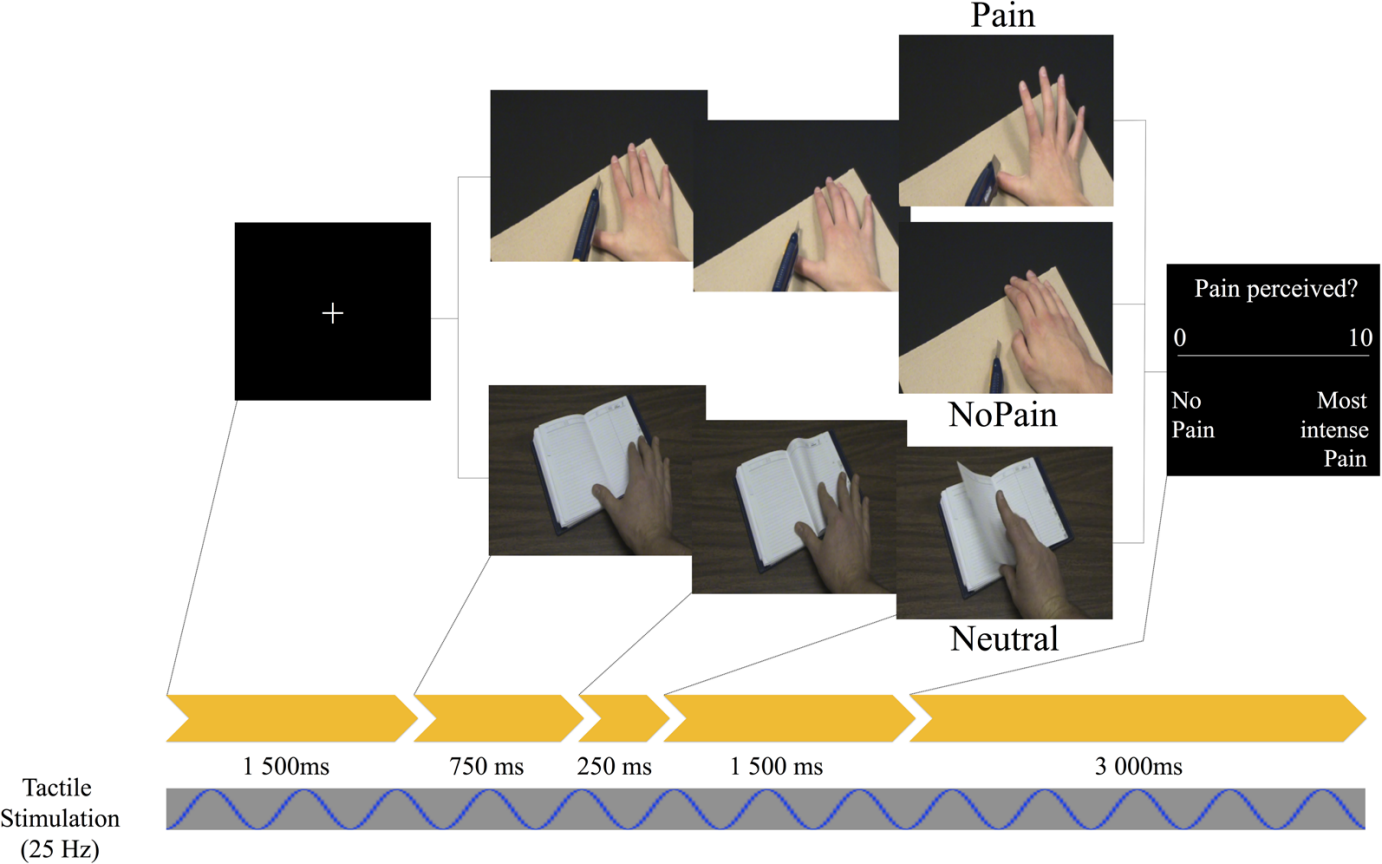
**Schematic of the experimental design depicting one trial**. Timing in ms (below yellow arrows) corresponds to the duration of each picture. A light repetitive stimulation at a frequency of 25 Hz was continuously applied to the palm of the right hand throughout data acquisition.

Stimuli consisted in a series of 30-color pseudo-dynamic pictures depicting hands of male and female adults in three different conditions: Painful, Non-Painful, and Neutral situations. Specifically, each stimulus involved a sequence of three visual static pictures presented in a short sequence (750 ms + 250 + 1500 = 2500 ms) to create the illusion of a movement (similar to the task described in Decety et al., 2009; see Figure 1). Different types of pain (mechanical and thermal) inflicted to the hands were displayed. The No Pain stimuli showed hands in visually similar situations as in the Pain condition but without the painful consequence [i.e., the 3rd frame differed; e.g., a knife on the finger (Pain) vs. a knife of the board (No Pain)]. Neutral stimuli showed hands in visually different situations devoid of any of the nociceptive elements found in the other two conditions (e.g., a hand grasping a set of keys or a tissue). We used a neutral condition to assess the possible priming effect of the nociceptive elements already found on the first picture of the Painful and Non-Painful conditions. The hands were shown from a maximum angle of 45° from the perspective of the observer, and all pictures were edited to show hands of same size and from approximately the same distance.

#### Tactile steady-state stimulation

Non-painful light repetitive (25-Hz) mechanical stimulations were continuously applied to the palm of the right hand using a custom-made vibrotactile stimulator similar to the one used in Voisin et al. (2011a,b). Compared to the previous stimulator, which targeted the ventral portion of the right index distal phalange, the one used in the current study stimulated the whole palm of the right hand.

#### EEG

EEG activity was acquired via 124 + 4 Ag/AgCl electrodes contacting the scalp surface by way of saline-soaked sponges (HCGSN, Electrical Geodesic Inc., Oregon). The amplifier system used for EEG recordings was an EGI GES250 system (Electrical Geodesic Inc., Oregon). The sampling rate was 500-Hz, with acquisition reference at the vertex. Electrodes impedances were kept below 50 kΩ.

#### Electromyographic activity

In order to ensure that the modulation in SG was not due to muscle contraction of the right hand, electromyographic activity (EMG) was recorded in all participants using Ag-AgCl surface electrodes placed in bipolar configuration over the First Dorsal Interosseus (FDI) muscle. EMG was amplified and band pass filtered (20–1000 Hz). The Acknowledge software (Biopac System) was used to acquire surface EMG and events code. Online visual inspection of the EMG output and inter-block feedback to participants ensured that this muscle stayed relaxed during EEG data acquisition and that the energy contained in the 25-Hz band frequency was produced by the stimulation.

### Procedure

Participants took part in a 60 min EEG session. They were seated in an armchair with their right arm on an arm-rest while watching a 20″ (~48 cm) LCD monitor positioned approximately at 85 cm. Stimuli were presented with a computer running the E-Prime software (Version 2.0, Psychology Software Tools, Inc.) to control the timing of the stimuli as well as the generation of event codes. Each trial began by a fixation cross (2500 ms), followed by a sequence of three static pictures (total time 2500 ms) successively presented, ending with visual rating scale (3000 ms) ranging from 0 (*no pain*) to 10 (*worst pain possible*) (see Figure 1). Subjects were told to refrain from blinking and performing head and jaw movements as much as possible during the presentation of fixation crosses and stimuli. After each scenario, participants were instructed to use the visual rating scale and verbally evaluate the level of pain that individuals would feel in each scenario via an intercom system as participants were seated in an audiometric room (Genieaudio Inc., Toronto). The experimental session consisted of six blocks of 30 trials lasting approximately 5 min each. The conditions were randomized and counter-balanced within each of the six blocks. Several practice trials were run prior to the experiment using other picture than those selected for the test trials. After the six experimental blocs, participants were asked to fill self-reported trait-empathy (IRI) and pain sensitivity (SPQ) questionnaires.

### EEG data preprocessing

All preprocessing was performed with the ELAB software developed at Centre de recherche en réadaptation et intégration sociale (CIRRIS) (Voisin et al., 2011a,b). ELAB is a series of Matlab routines allowing the control of the ELAN-Pack software developed at INSERM Brain Dynamics and Cognition team of the Lyon Neuroscience Research Center (Aguera et al., 2011). Raw data was first parsed into event, and indexed according to the type of the stimuli. Two faulty electrodes caused unreliable signal across all subjects and were removed from the analysis [electrodes 83 and 114 in the EGI system (HCGSN) corresponding to T10 and O2 in the 10–20 systems]. Then, a first rejection criterion was applied on the basis of any rating for a Painful stimulus <1, for a Neutral or Non-Painful stimulus >1 led to the rejection of the related-event, to ensure that further analyses would be made only on task-relevant data. Inspection of the data distribution enabled the selection of a series of criteria meant to detect blinks, muscle activity, and fast baseline shift. They were set to reject any sample that fell within 100 ms of one of these events: (1) the scalp potential exhibited variation over 200 μV within a 200 ms time window in the same electrode channel; (2) the energy content was more than 500 μV^2^ in the 60–100 Hz band in the same electrode channel; (3) the scalp potential exhibited variation over 50 μV within a 10 ms time window in the same electrode channel; (4) the energy content was more than 1500 μV^2^ in the 23–27 Hz band in the same electrode channel. The remaining data consisted of 77% of the original set. This remaining signal was submitted to a spherical spline interpolation process (Perrin et al., 1989), using Tikhonov regularization in order to reduce sensitivity to noise (Babiloni et al., 1998). This procedure allows the reconstruction of the signal of a noisy electrode based on the signal of the noise-free electrodes. Notably, this process poses a specific challenge as the rejected samples can be broadly distributed across time and electrodes so that a proper reconstruction has either to reject all samples each time a faulty electrode is found, or to reject all electrodes that included at least one rejected sample. Thus, any fixed method would have led to rejecting a large portion of the data. ELAB software allowed circumventing this problem by selecting, automatically for each trial, the set of electrodes that should enter the interpolation process so as to maximize the number of valid samples used. In the present experiment, the best solutions used a mean of 70% of the original samples (intersubject variability 48–93%) to reconstruct the signal. More precisely, the interpolation process was based on average on 77% of the 124 electrodes positioned on the scalp (intersubject variability 47–97%) and on average, 91% of the time bins (intersubject variability 68–99%). Once the signal was split-transformed, it was convoluted with complex Gaussian Morlet's wavelets (Tallon-Baudry and Bertrand, 1999) intended to extract the energy in the 25 Hz range (omega, 24–26; sigma, 3.6), representing the energy band in which the cortical response to the somatosensory stimulation used in the current study should be condensed. Mean 25 Hz range energy during the fixation cross (1000 ms before stimulus onset) was then computed, and any trial in which the baseline mean energy dispersion was over two standard deviation from the whole bloc mean energy was rejected (an average of one trial was rejected per subject, max rejection was two trials). No subject was rejected from analysis.

### Statistical analyses

#### Behavioral data

Differences on mean pain ratings between conditions and groups were computed using a 3 (Conditions: Pain vs. NoPain vs. Neutral) × 2 (Groups: LSRP_high vs. LSRP_low) repeated measures analysis of variance (ANOVA). The relation between pain ratings and psychopathy scores (LSRPtotal, PP1, and PP2) were explored with Pearson correlations. In order to assess between group differences on the independent subscales of self-reported empathy (IRI), four independent sample *t*-tests were realized. Pearson correlations were then used to determine the relationship between empathy and psychopathy scores. Finally, group differences on pain sensitivity discrimination P(A) and bias scores (B) of the SPQ were tested with two independent sample *t*-tests.

#### EEG

A similar procedure as in Voisin et al. (2011a) was used to analyze the SSSR. First, epochs in all three conditions were averaged to delineate the regions of interest (ROI) for each group. Subtraction maps were then created by subtracting the baseline period (−1000:0 ms, the cross duration) from the first two pictures period (0:1000 ms). This procedure allows the visual identification of the electrodes in which SG was showing the greatest modulation during the first two pictures in comparison to baseline (fixation cross), for all conditions. Note that the maps were created from (1000 ms) time bins and statistical analyses were then all realized with more circumscribed 200ms time bins to increase accuracy. This initial analysis identified the following ROI electrodes [parietal electrodes 66, 67, and 71 in the EGI system (HCGSN) corresponding to P3 in the 10–20 systems] on which the remaining of the analyses was done.

Prior to test the *non-specific initial gating* (i.e., not imputable to the observation of pain), defined as the mean energy (mA/m3) difference between Fixation Cross Baseline (−200:0 ms) and Gating period (600:800 ms) (see Voisin et al., 2011a), Cross Baseline stability was verified using a 3 (Conditions: Pain vs. NoPain vs. Neutral) × 2 (Groups: LSRP_high vs. LSRP_low) repeated measures ANOVA. To investigate initial gating effect, mean energy during Gating period (600:800 ms) and Cross Baseline (−200:0 ms) were compared for each condition using simple *t*-tests against H_0_ (i.e., absence of gating). Second, *pain anticipation* [(Pain = Nopain) > Neutral] was tested by comparing mean energy ratios between the three experimental conditions during the Gating period (600:800 ms) with a one-way repeated measures ANOVA [Gating period × Conditions (3: Pain vs. NoPain vs. Neutral)]. Third, Pain Gating was assessed using ratios [(Second Picture Baseline - 3rd Picture Pain Gating)/Second Picture Baseline] by comparing painful and non-painful conditions for each participant in order to verify the specific modulation imputable to the onset of painful conditions using a 2 (Conditions: Pain vs. NoPain) × 2 (Groups: LSRP High vs. LSRP Low) repeated measures ANOVA. The 3rd Picture Gating period (1100:1700 ms) was divided in three (200 ms) time bins. Separated analysis was performed on each time bin. All the analyses were done with an alpha level set at 0.05 and corrected with Bonferroni procedure for multiple comparisons.

#### Mediation analysis

As sensorimotor resonance was previously found to be positively associated with scores on Coldheartedness subscale (Fecteau et al., 2008), which reflect a lack of empathy and sensibility toward others, and conversely positively correlated with trait-empathy (Avenanti et al., 2009), we sought to explore the indirect effect of primary psychopathy on the relationship between self-reported empathy and SG to pain. This was tested using the bootstrapping method developed by Preacher and Hayes (2004, 2008; see Simple Mediator model). This non-parametric method overcomes limitations of the Baron and Kenny's (1986) causal steps and Sobel's test that are conservative and not likely to detect indirect effects in smaller samples. Moreover, this method has the benefit of not assuming normality of the sampling distribution of the indirect effect and allows testing of mediating effect (Preacher and Hayes, 2004). The SPSS macro developed by Preacher and Hayes (2008) provides the strength of direct effects of independent and mediating variables. Preacher and Hayes (2004) also stated that it is possible to find a significant indirect effect even if there is no evidence of a significant total effect (path c, see Figure 7). Point-estimate of the indirect effect and 95% bias corrected confidence intervals (BC) were computed based on a 5000 bootstrap resample. In order to conclude for the presence of a mediating effect, the 95% BC confidence interval must not include zero, thus suggesting that the value of the indirect effect is significantly different from zero. Note that the relatively small sample in the current study suggests caution in drawing inference from the mediation analysis.

## Results

### Behavioral results

**Figure 2:**
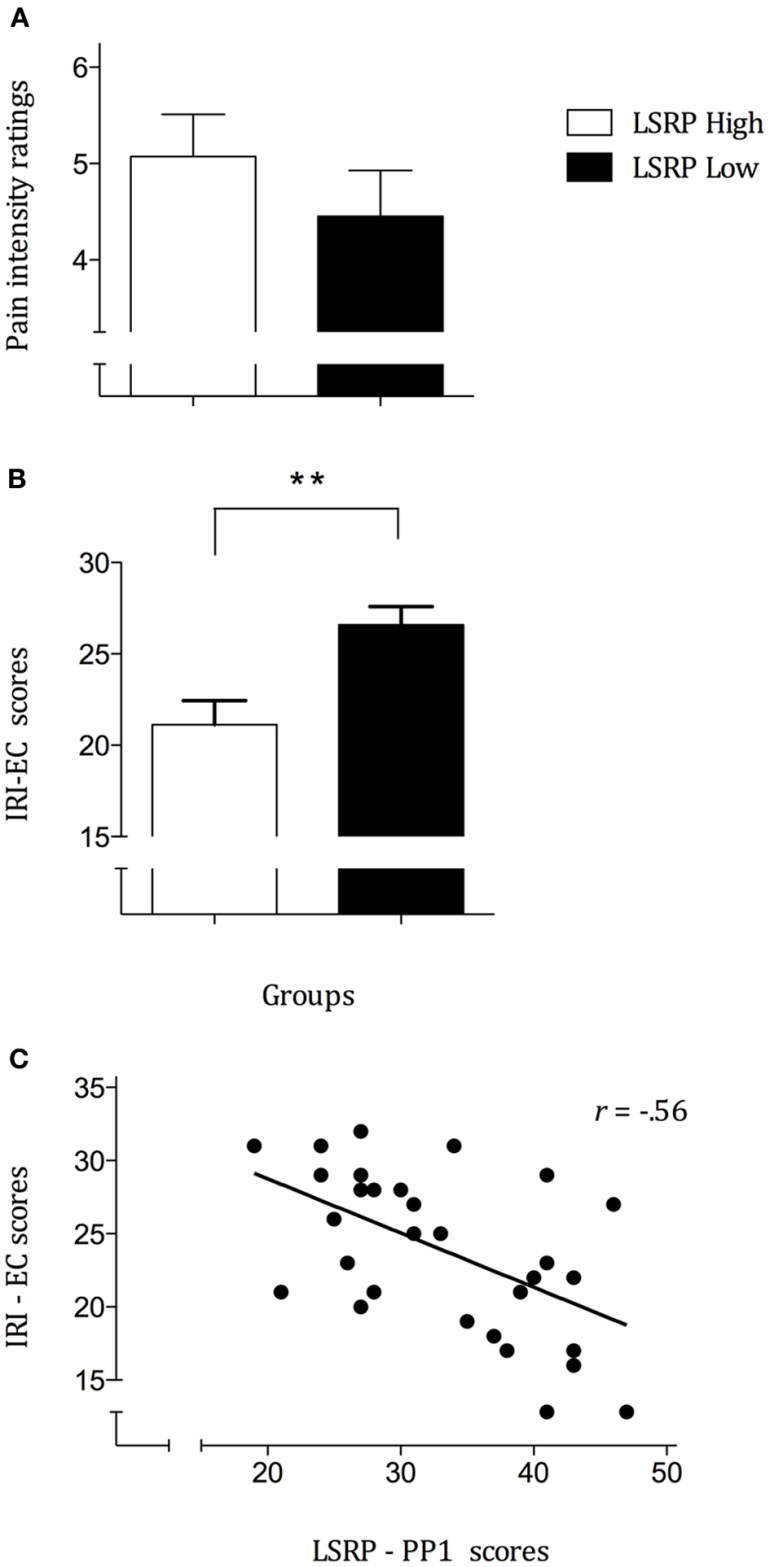
**(A)** Mean pain ratings for each group indicating an absence of significant difference (*p* = 0.35) between LSRP_High and LSRP_Low; **(B)** Self-reported Empathic Concern (EC) scores significantly differed between LSRP_High and LSRP_Low group; **(C)** Negative correlation between primary psychopathy (PP1) scores and empathic concern scores. ^**^p < 0.01.

Analyses performed on pain intensity ratings confirmed the expected significant effect for the main effects of Condition [*F*_(1, 28)_ = 160.7, *p* < 0.001] whereas no significant effect was observed for Group [*F*_(1, 28)_ = 0.21, *p* = 0.657] nor their interaction [*F*_(1, 28)_ = 1.19, *p* = 0.283]. *Post-hoc* pair comparisons showed that mean pain ratings for painful scenarios (4.9 ± 0.362) differed significantly from non-painful (0.002 ± 0.001; *p* < 0.001) and neutral scenarios (0.03 ± 0.021; *p* < 0.001) whereas no difference has been found between the latter two (*p* = 0.143). As illustrated in Figure 2A, between-group analyses showed no significant differences for the mean ratings in the pain condition (LSRP_High: 5.1 ± 0.441; LSRP_Low: 4.5 ± 0.473). To compare the differences between LSRP_Low and LSRP_High participants on trait empathy, independent *t*-test on each IRI subscale were used and revealed no significant between-groups difference on three of the four subscales [PT: *t*_(29)_ = 2.3, *p* = 0.142; F: *t*_(29)_ = 0.562, *p* = 0.47; D: *t*_(29)_ = 0.962, *p* = 0.344]. Figure 2B shows the only subscale (EC) for which a significant difference between both groups was found [LSRP_Low: 19.6 ± 3.7; LSRP_High: 14.1 ± 4.9; *t*_(29)_ = 10.9, *p* = 0.003]. Over all participants, the correlations showed a significant negative relationship between the EC subscale and the LSRP_total score (*r* = −0.561, *p* = 0.001), as well as between the EC subscale and the PP1 subscale (*r* = −0.560, *p* = 0.001; Figure 2C) indicating an inverse relationship between affective empathy and psychopathic traits. Between-group analyses on the pain sensitivity responses indicated no significant differences for the discrimination [PA: *t*_(29)_ = 0.21, *p* = 0.668] nor the bias scores of the SPQ [B: *t*_(29)_ = 1.9, *p* = 0.184].

### EEG results

#### General gating effect

**Figure 3:**
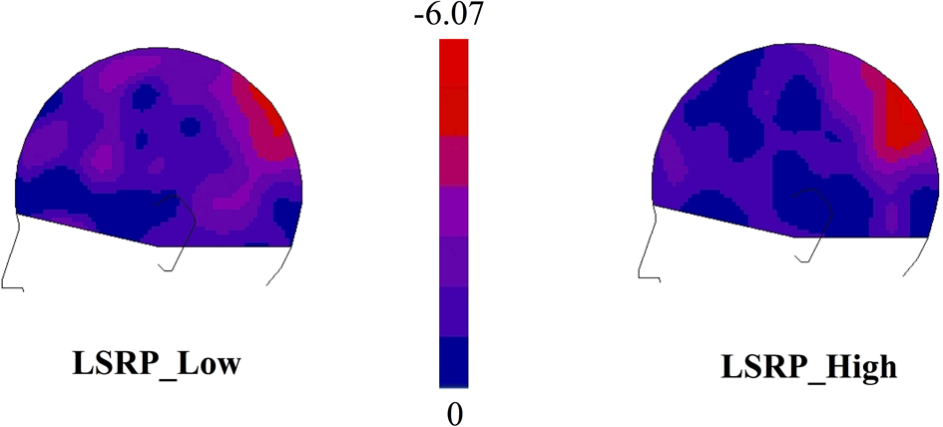
**Subtraction maps created to identify the ROI electrodes [66-67-71, in the EGI system (HCGSN) corresponding to P3 in the 10–20 systems] in which the somatosensory gating (SG) was showing the greatest modulation during the first two pictures (0:1000 ms) in comparison to the Cross Baseline (−1000:0 ms)**.

EEG data showed that the maximal change in SG during the visual presentation of the first two stimuli was over the parietal cortex controlateral to the stimulated hand for both experimental groups. As illustrated in Figure 3, subtraction maps (First two pictures − Fixation cross) indicated a strong decrease in left caudal part of the parieto-central region [electrodes 66, 67, 71 in the EGI system (HCGSN) corresponding to P3 in the 10–20 systems] for both groups. A decrease in the 25 Hz energy band was also found in the same region during the presentation of static stimuli depicting hand in painful and non-painful situations in previous EEG studies using a similar protocol (Voisin et al., 2011a,b,c). Statistical analyses were then restricted to this region specifically showing SG.

In order to assess baseline stability during the Cross Baseline period (-200:0 ms) prior to the first picture onset, a 3 (Conditions: Pain vs. NoPain vs. Neutral) × 2 (Groups: LSRP_Hihg vs. LSRP_Low) repeated measures ANOVA was conducted. No significant effect was observed neither for main effects of Condition [*F*_(1, 28)_ = 0.06, *p* = 0.812] or Group [*F*_(1, 28)_ = 1.71, *p* = 0.201] nor their interaction [*F*_(1, 28)_ = 0.31, *p* = 0.583], reducing the chance that the Cross Baseline period could be the source of subsequent differences.

**Figure 4:**
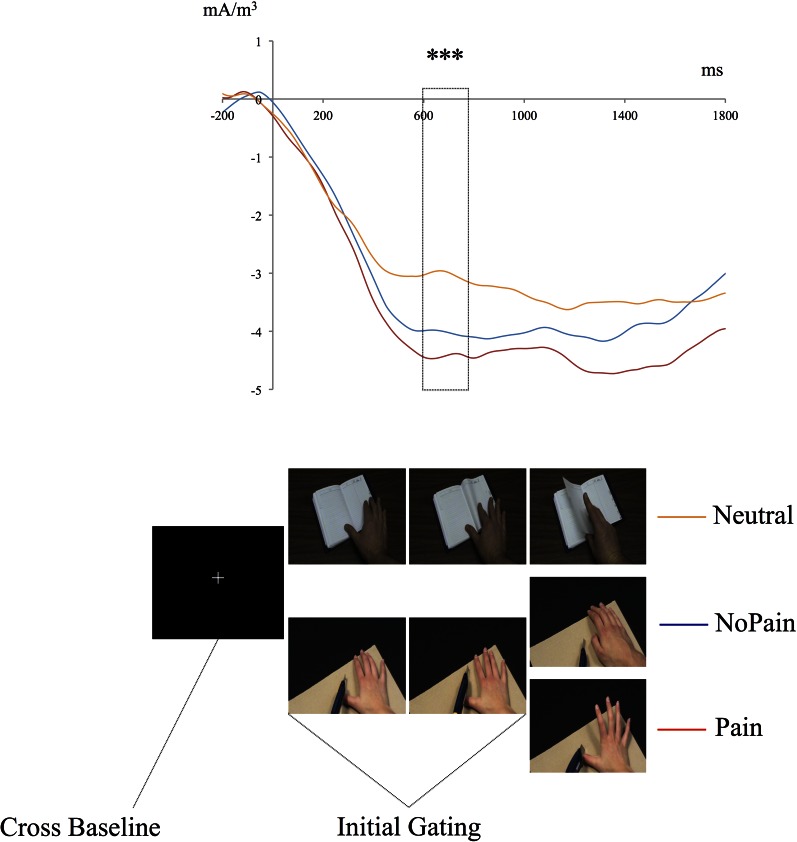
**Time course of the mean energy (mA/m^3^) of the somatosensory steady-state response (SSSR) during the presentation of the pseudo-dynamic stimuli**. The mean energy of the somatosensory gating (SG) during the first two pictures [initial gating (600:800 ms)] was significantly different from mean energy during the Cross Baseline (−200–0 ms) for each condition and for all participants. The magnitude of the SG during the initial gating (600:800 ms) was significantly greater in the Pain and NoPain conditions compared to the Neutral condition. ^***^p < 0.001.

Figure 4 shows the decrease in the 25 Hz energy band irrespective of the experimental conditions stabilizing 600–800ms after the first picture onset. To investigate this general gating effect, mean energy ratios during Gating period (600:800 ms) and Cross Baseline (−200:0 ms) were compared for each condition using simple *t*-tests against H_0_ which is the absence of gating (ratio = 0). On average, modulation amplitude reached.19, corresponding to 19% of Cross Baseline raw amplitude. Contrasts between Baseline and Gating period were all statistically significant [NoPain: ratio = 0.21 ± 0.03; *t*_(29)_ = 5.9, *p* < 0.001; Pain: ratio = 0.23 ± 0.02; *t*_(29)_ = 7.8, *p* < 0.001; Neutral: ratio = 0.14 ± 0.02; *t*_(29)_ = 4.0, *p* < 0.001], confirming that the observation of the stimuli depicting hands, irrespective of the condition, triggered changes in sensory processing of somatic information in the observer.

#### Pain anticipation effect

To assess the possible effect of pain anticipation, mean energy ratios were compared between the three experimental conditions during the Gating period (600:800 ms). A significant effect was found for Conditions [*F*_(1, 28)_ = 6.8, *p* = 0.014] but not for Groups [*F*_(1, 28)_ = 1.3, *p* = 0.262]; the interaction was not significant [*F*_(1, 28)_ = 1.2, *p* = 0.294]. Paired comparisons for Conditions showed that Neutral significantly differed from Pain (*p* = 0.013) and NoPain (*p* = 0.043) whereas the latter two did not (*p* = 0.891).

#### Pain observation effect

In order to assess Pain Gating, a baseline period was set during the second picture (800:100 ms) for the Pain and NoPain conditions. The stability of this baseline was tested by comparing mean energy for both condition using a 2 (condition: Pain vs. NoPain) × 2 (groups: LSRP Low vs. High) repeated measures ANOVA. No significant effect was observed for the main effects of Condition [*F*_(1, 28)_ = 0.27, *p* = 0.612] or Group [*F*_(1, 28)_ = 2.6, *p* = 0.121] nor their interaction [*F*_(1, 28)_ = 0.05, *p* = 0.833], confirming that Second Picture Baseline would not account for later differences.

**Figure 5:**
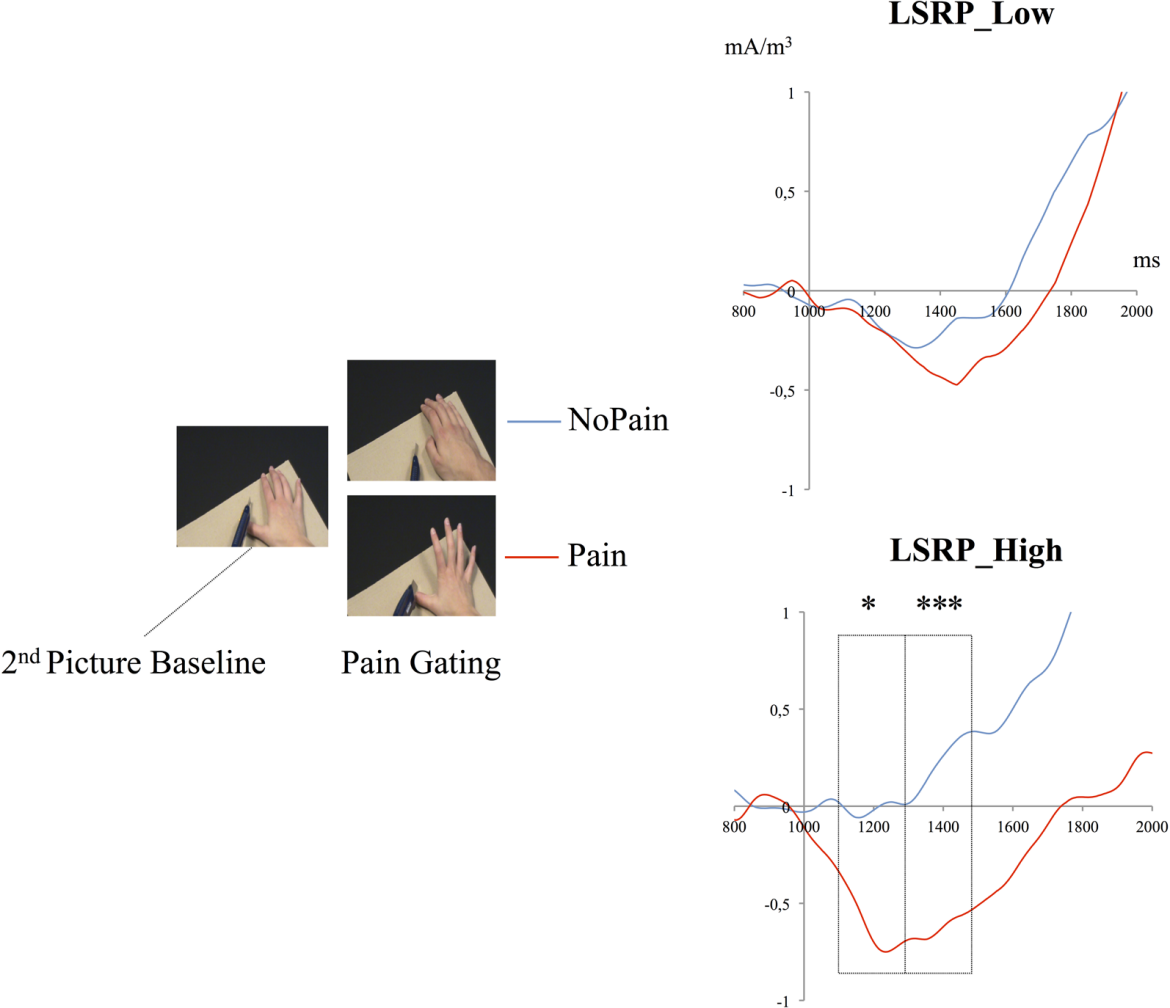
**Time course of the mean energy (mA/m^3^) of the SG during the presentation of the third picture (i.e., the picture where the painful contact occurred or not)**. The mean energy ratios during the (1300:1500 ms) and (1500:1700 ms) periods were significantly different from that of the Second Picture Baseline (800:100 ms) only in the LSRP_High group. ^*^p < 0.05, ^***^p < 0.001.

Mean energy ratios were subsequently compared between Pain and NoPain conditions for both groups during the third picture period (1100:1700 ms) through three (200 ms) time bins (see Figure 5). 2 (condition: Pain vs. NoPain) × 2 (groups: LSRP Low vs. High) repeated measures ANOVA were conducted on the same three time bins. During the (1100:1300 ms) period, main effects of Condition [*F*_(1, 28)_ = 3.8, *p* = 0.063] and Group [*F*_(1, 28)_ = 2.8, *p* = 0.114] did not reach statistical significance. Still the effect of interaction between both Condition and Group was significant [*F*_(1, 28)_ = 4.8, *p* = 0.042]. *Post-hoc* analyses revealed a significant difference between Pain and NoPain Conditions only for the LSRP_High (*p* = 0.014; LSRP_Low: *p* = 0.863). Throughout the (1300:1500 ms) period, no significant effect was observed for main effects of Condition [*F*_(1, 28)_ = 2.1, *p* = 0.163] or Group [*F*_(1, 28)_ = 3.5, *p* = 0.074]. However, a significant interaction was found [*F*_(1, 28)_ = 6.2, *p* = 0.024]. *Post-hoc* analyses in each group showed a significant difference between Pain and NoPain Conditions for the LSRP_High group (*p* = 0.001), but not for the LSRP_Low group (*p* = 0.563). For the (1500:1700 ms) period, no significant effect was found for main effects of Conditions [*F*_(1, 28)_ = 0.8, *p* = 0.382] or Group [*F*_(1, 28)_ = 3.8, *p* = 0.074] nor their interaction [*F*_(1, 28)_ = 3.2, *p* = 0.081].

### Correlation between the behavioral and the EEG results

**Figure 6:**
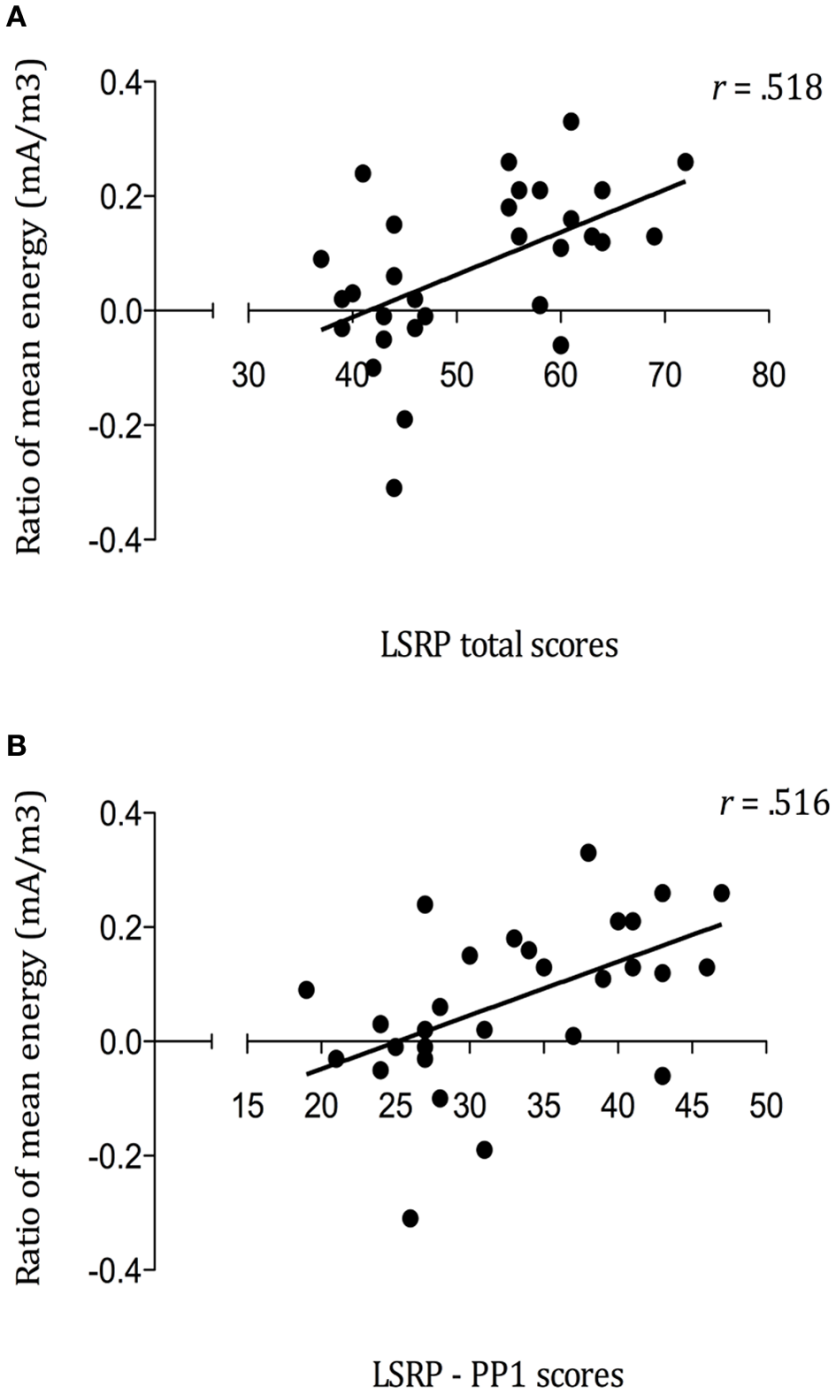
**Positive correlations between mean energy (mA/m^3^) ratios during Pain Gating (1300–1500ms) and (A) self-reported psychopathy total scores (*p* = 0.003); (B) primary psychopathy (PP1) subscale scores (*p* = 0.004)**.

In order to assess the linear dependence between the modulation of SG during pain observation and psychopathic traits, Pearson correlations were used. The analyses performed on the mean energies ratios for the pain picture [Second Picture Baseline (800:100 ms) − Third Picture maximal Gating (1300–1500 ms)/Second Picture Baseline] pointed out some positives associations with LSRP scores. As illustrated in Figure 6, strong positive correlations were found between SG during pain observation and LSRP_Total scores (*r* = 0.518, *p* = 0.003; Figure 6A), and PP1 scores (*r* = 0.516, *p* = 0.004; Figure 6B). However, the relationship between SG and the PP2 scores did not reach statistical significance (*r* = 0.29, *p* = 0.122). No significant correlation was found between SSSR and any of the IRI subscales (PT: *r* = 0.15, *p* = 0.431; F: *r* = −0.06, *p* = 0.763; EC: *r* = −0.21, *p* = 0.284; D: *r* = 0.03, *p* = 0.861). Finally, no significant relationship was found between SG during pain observation and Pain ratings (*r* = 0.11, *p* = 0.562).

### The indirect effect of primary psychopathy

**Figure 7:**
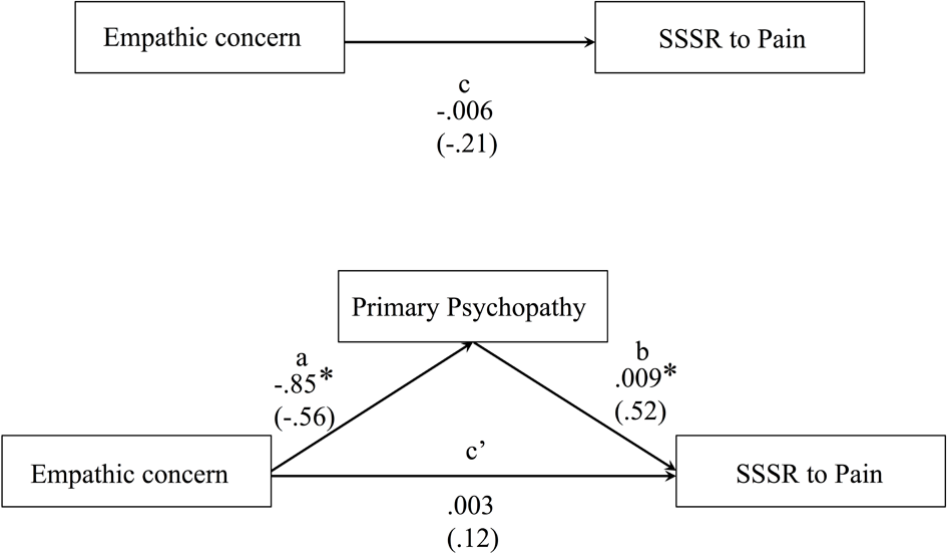
**Illustration of the direct effects of the bootstrap mediating model predicting SG to pain observation (*N* = 30) using the 5000 bootstrap samples**. Path values represent both unstandardized regression coefficients (bold) and standardized regression coefficients (in brackets). ^*^p < 0.05.

Figure 7 presents the results of the mediation model of direct and indirect effects. The model aimed at testing the interplay between empathy and psychopathy during somatosensory resonance. The results indicated that the total effect of EC on SG to pain (path c) remained non-significant but changed its direction (path c') after introducing primary psychopathy as a mediator. Point-estimate of the indirect effect of EC on SSSR to pain through primary psychopathy was −0.0091 with a 95% BC confidence interval of −0.0200 to −0.0039. Because zero was not in the confidence interval, we can conclude that there is a significant indirect effect [R^2^ for the mediating model = 0.277, *F*_(2, 27)_ = 5.16, *p* = 0.013], suggesting that primary psychopathy is a mediator of EC predicting SG to the pain of others. This suggests that psychopathic traits in community individuals contribute to the relation between the affective empathy and somatosensory resonance during pain observation in others.

## Discussion

The goal of this study was to assess changes in somatosensory processing during pain observation in a group of male college students with respect to self-reported empathy and psychopathic traits. Generally, the observation of pseudo-dynamic stimuli depicting hands in Painful and Non-Painful scenarios produced a modulation of the SG response to a mechanical stimulation of the right hand in both high and low psychopathic traits groups. Modulation of the SG was maximal in a parieto-central region contralateral to the stimulated hand. This corroborate previous finding using a similar design (Voisin et al., 2011a) and parallel results showing that observing the body improves tactile performance and modulates SEP (e.g., Taylor-Clarke et al., 2002; Morrison et al., 2007; Cardini et al., 2011). Interestingly, SG specific to pain observation was statistically significant only for the LSRP_High group. Overall, this SG was also positively correlated with affective and interpersonal aspect of psychopathy. Moreover, EC scores were significantly lower in this group compared to LSRP_Low, suggesting that increase somatosensory resonance to other's pain is not exclusively explained by components of affective empathy and may be linked to other personality traits, such as psychopathy. In fact, results from the mediation analysis indicated that primary psychopathy might play a role of mediator in the relation between EC and SSSR to pain.

### Self-reported empathy negatively correlated with psychopathic traits

Our behavioral results showed that LSRP_High and Low groups did not differ in their subjective evaluation of pain intensity. This result seems to be in line with previous works reporting that both healthy and conduct disorder adolescents displaying psychopathic traits judged painful stimuli as similarly more painful (Decety et al., 2009) and that pain ratings in juvenile offenders characterized by high and low callous-unemotional traits did not differ (Cheng et al., 2012). The significant difference found between High and Low LSRP groups on IRI-EC subscale adds to the inconsistent findings regarding differences in self-reported empathy among psychopathic and their respective comparison groups. If negative correlations between self-reported empathy and psychopathic traits have been more consistently reported (Sandoval et al., 2000; Jolliffe and Farrington, 2004; Mahmut et al., 2008), some studies have failed to show differences on IRI subscales when comparing psychopathic offenders with non-psychopathic offenders with antisocial personality disorder and community samples (Book and Quinsey, 2004; Dolan and Fullam, 2004). Indeed, psychopathy and antisocial personality disorder might be conceived as dimensional constructs (Marcus et al., 2006), hence reducing the possibility to found between group differences on empathy. Besides, the use of self-report empathy with correctional sample may offer limited efficacy as deception, manipulation and grandiose sense of self-worth are at the core of psychopathic manifestation. In the current study, the significant difference found on IRI-EC subscale might be attributed to the composition of the non-forensic sample, as low score on antisocial deviance were found in both groups. The absence of between-group difference in PT is also congruent with current conceptions that psychopathic individuals are seen as having a reduced sensibility to other's distress instead of an incapacity to adopt the psychological perspective of others (Dolan and Fullam, 2004; Blair, 2006).

### The somatosensory gating was stronger when pain was anticipated

The results of the present study also showed that the increase in the magnitude of SG was more important in the first two pictures for Pain and NoPain conditions compared to Neutral condition. This suggests that contextual dependent effect of the nociceptive elements found in the former conditions might account for the difference in the mean levels of energy. They also support the assumption that whenever our attention is directed to the somatic cause of pain (Bufalari et al., 2007; Lamm et al., 2007), somatosensory processes are engaged by the observer, allowing him or her to create a cerebral representation of others' painful experience by assigning a *quantitative sense of pain* (Keysers et al., 2010). These results might also be explained by possible pain anticipation. It was previously shown that anticipation of pain in others triggered fear-potentiated startle reflex (Caes et al., 2012) thus potentially modulated the SG to pictures containing nociceptive components. In addition, the study of Caes et al. (2012) demonstrated that startle reflex was blunted in participants depicting higher psychopathic traits. Yet, the current study did not show a significant difference between high and low psychopathic traits group on SG to pain anticipation. The stronger SG found during the first two pictures in which the nociceptive component was displayed compared to neutral pictures indicated a specific change in somatosensory activity during pain anticipation.

### Psychopathic traits facilitated pain-related somatosensory resonance

To our knowledge, this is the first study to show that pain observation modulates SG to a greater extend in male college students with high scores on self-reported psychopathy compared to participants with low scores. Other studies have, however, accumulated evidence supporting enhanced somatosensory response to other's pain in male adolescent with high psychopathic traits (Decety et al., 2009; Chen et al., 2012). Specifically, adolescents with conduct disorders and psychopathic traits showed greater sensorimotor resonance for neural response to pain perception compared to healthy adolescents (Decety et al., 2009). Furthermore, young offenders with high callous-unemotional traits showed stronger *mu* suppression (10 Hz) compared to the low ones during pain observation (Cheng et al., 2012). Together, these results are in accordance with our findings, suggesting a greater sensorimotor resonance to other's pain in samples characterized by a reduced capacity for empathy and compassion toward other's distress. This speaks for a more complex link between empathy for pain and resonance than the direct relationship previously proposed, and argue for the contribution of regulation mechanisms allowing prosocial reactions (Decety and Jackson, 2004; Singer et al., 2004; Vachon-Presseau et al., 2012).

Our results also parallel findings from previous studies reporting that SEP elicited by tactile stimulation were modulated by negative emotional stimuli in healthy adults (Montoya and Sitges, 2006) and that the aversion felt during observation of others' pain is negatively correlated with the magnitude of sensorimotor response to others' pain (Avenanti et al., 2009). This is also in line with findings from Decety et al. (2009) who showed greater responses in regions dedicated to affective and sensory components of pain perception in conduct disorders adolescent with psychopathic traits. Specifically, connectivity analysis demonstrated stronger activation of amygdala and striatum together with reduced response in orbitofrontal cortex, suggesting that seeing pain in others did not generate distress in these adolescents but could have led to pleasant feelings. All together, these results suggest that the presence of high psychopathic traits can attenuate the effect of negative emotional arousal caused by the observation of pain in others, thus increasing attention to the sensory components of the stimuli displayed.

Another interesting result consists in the positive correlations found between SG during pain observation and LSRP_Total scores, as well as between SG to pain observation and PP1 subscale scores, which support and extend the findings of Fecteau et al. (2008). As previously demonstrated, participants who scored higher on a specific psychopathic traits subscale (Coldheartedness) showed greater corticospinal inhibition (Fecteau et al., 2008). Interestingly, this subscale measures the absence of deep feeling of guilt and empathy, reflecting the tendency to lack of caring for others (Lilienfeld and Andrews, 1996), all referring to the affective and interpersonal dimension of psychopathy, namely primary psychopathy. However, the negative correlation between empathic concern (IRI-EC) and SG to pain observation did not reach significance. Still, a negative relationship was confirmed between IRI-EC and the PP1 subscale. The fact that the correlations found between the SSSR modulation to pain and both LSRP_total and PP1 subscale are similar (total: *r* = 0.518; PP1: *r* = 0.516) and the absence of significant relation with the PP2 subscale is interesting. These findings suggest that affective and interpersonal aspects of psychopathy constituted the principal factor explaining the modulation of the somatosensory gating. As it might be expected in a community sample study, the PP2 scores resulting from the evaluation of social deviance were low in both groups but still differed significantly; the scores were not comparable to those of incarcerated samples. Nevertheless, results from a community sample indicated that the PP1 factor is more related to high narcissism and prototypical psychopathy compared to the PP2 factor, which tend to be associated with a broad range of personality disorders (Miller et al., 2008).

### Primary psychopathy mediated the link between empathy and somatosensory resonance

Results from the Simple Mediator model confirmed the mediating role of primary psychopathy on empathic concern in predicting SG to pain observation. One plausible hypothesis that could account for the absence of significant direct relation between empathic concern and SG to pain observation is the interaction of the suppressor effect revealed by the negative correlation between empathic concern and primary psychopathy with the facilitator effect of primary psychopathy on SG to pain observation. The findings from the mediation analysis could help interpreting the divergent relationship found between enhanced sensorimotor resonance and trait-empathy (Avenanti et al., 2009), as well as between resonance and coldheartedness traits (Fecteau et al., 2008). The results show that psychopathic traits mediated the relation between empathic concern and SG, arguing against the assumption of a straight path between sensorimotor resonance and empathy. This finding is important because it suggests that psychopathic traits in healthy individuals could explain the great inter-individual variability in sensory resonance when decoding pain in others. Further studies will need to dissect the affective and interpersonal qualities that might best contribute to the mediating role of primary psychopathy.

### Limitations and further studies

Some limitations can be pointed out with respect to the proposed interpretation of the findings. First, the use of somatosensory steady-state and time-frequency analysis offer more precision in the frequency domain compared to event-related potential (ERP) and peak to peak analysis but this come with a cost in terms of temporal resolution, as reflected by the use of relatively long time bins (200 ms) in the analyses. Subtle changes in SG relative to temporal dynamics of pain perception might thus have been missed with this method. For instance, the effect of psychopathic traits on pain anticipation was previously shown in a study using ERP with young offenders by assessing early negative arousal (Cheng et al., 2012). Second, the use of extreme scores on the LSRP to form experimental groups may have contributed to the absence of significant SG to pain observation in the LSRP_Low group. Even if this remains speculative, some personality traits and/or emotional factor such as higher negative arousal than individuals in the mid-range of LSRP scores could account for the absence of significant SG during pain observation in the LSRP_Low group. However, mean scores on the PD subscale did not significantly differ between groups and the direction of the relation between negative arousal and sensorimotor response to other's pain needs to be clarified (Meng et al., 2013). Therefore, the present results should be interpreted with regards to the direction of the effect instead of its magnitude. Indeed the more robust outcomes, explaining the largest proportion of the variance, were the correlation between LSRP_total/PP1 scores and SG to pain observation suggesting that a dimensional approach might be more appropriate to understand somatosensory resonance with respect to psychopathic traits.

In the current study, the correlation between pain ratings and SG to pain observation was not statistically significant. However, prior studies on pain perception have shown significant positive correlations between sensorimotor processing and evaluations of pain intensity (e.g., Avenanti et al., 2005; Bufalari et al., 2007; Valeriani et al., 2008; Betti et al., 2009). This suggest a multifaceted relationship between sensorimotor resonance and evaluation of others' bodily feelings, suggesting that somatosensory response may not be exclusively related to the intensity of the pain perceived but also to the arousal generated by the stimuli (Bolognini et al., 2013). Future studies will need to clarify the likely interaction of affective arousal on somatosensory processing.

## Conclusion

This study demonstrated that observing pain in others triggered somatosensory gating to a greater extends in college male students with high psychopathic traits compared to students with low psychopathic traits. It provides additional evidence on the relationship between personality traits associated with affective and interpersonal dimensions of psychopathy and somatosensory resonance to other's pain. The mediation effect found for psychopathic traits thus gives insight into the complex relationship between trait empathy and somatosensory processing of other's pain. The current study also contribute to extend the growing body of literature on psychopathic correlates in non-incarcerated samples trying to depict a sharper representation of the affective-related alterations observed in these individuals, thus supporting a dimensional approach of psychopathy.

### Conflict of interest statement

The authors declare that the research was conducted in the absence of any commercial or financial relationships that could be construed as a potential conflict of interest.
